# The Effect of TGF-β1 Reduced Functionality on the Expression of Selected Synaptic Proteins and Electrophysiological Parameters: Implications of Changes Observed in Acute Hepatic Encephalopathy

**DOI:** 10.3390/ijms23031081

**Published:** 2022-01-19

**Authors:** Mariusz Popek, Bartosz Bobula, Karolina Orzeł-Gajowik, Magdalena Zielińska

**Affiliations:** 1 Department of Neurotoxicology, Mossakowski Medical Research Institute, Polish Academy of Sciences, 5 Pawińskiego Str., 02-106 Warsaw, Poland; korzel@imdik.pan.pl; 2 Department of Physiology, Maj Institute of Pharmacology, Polish Academy of Sciences, 12 Smętna Str., 31-343 Krakow, Poland; bobula@if-pan.krakow.pl

**Keywords:** transforming growth factor β1, synaptic proteins, glutamatergic transmission, LTP, acute liver failure, blood–brain barrier

## Abstract

Decreased platelet count represents a feature of acute liver failure (ALF) pathogenesis. Platelets are the reservoir of transforming growth factor 1 (TGF-β1), a multipotent cytokine involved in the maintenance of, i.a., central nervous system homeostasis. Here, we analyzed the effect of a decrease in TGF-β1 active form on synaptic proteins levels, and brain electrophysiology, in mice after intraperitoneal (ip) administration of TGF-β1 antibody (anti-TGF-β1; 1 mg/mL). Next, we correlated it with a thrombocytopenia-induced TGF-β1 decrease, documented in an azoxymethane-induced (AOM; 100 mM ip) model of ALF, and clarified the impact of TGF-β1 decrease on blood–brain barrier functionality. The increase of both synaptophysin and synaptotagmin in the cytosolic fraction, and its reduction in a membrane fraction, were confirmed in the AOM mice brains. Both proteins’ decrease in analyzed fractions occurred in anti-TGF-β1 mice. In turn, an increase in postsynaptic (NR1 subunit of N-methyl-D-aspartate receptor, postsynaptic density protein 95, gephyrin) proteins in the AOM brain cortex, but a selective compensatory increase of NR1 subunit in anti-TGF-β mice, was observed. The alterations of synaptic proteins levels were not translated on electrophysiological parameters in the anti-TGF-β1 model. The results suggest the impairment of synaptic vesicles docking to the postsynaptic membrane in the AOM model. Nevertheless, changes in synaptic protein level in the anti-TGF-β1 mice do not affect neurotransmission and may not contribute to neurologic deficits in AOM mice.

## 1. Introduction

Acute liver failure (ALF) is characterized by rapid loss of liver function, which may result in a neurologically specific syndrome defined as hepatic encephalopathy (HE), encompassing symptoms related to systemic and central disturbances [1,2]. Thrombocytopenia is clinically the most frequent and common complication observed in patients with ALF. It is assumed that a decrease in platelet count can define the severity of liver dysfunction. Decreasing platelet amount signals systemic complications, contributing to HE-related alterations, and the need for a liver transplant. Importantly in the context of our study, platelets are the main source of transforming growth factor (TGF-β1), a cytokine exerting regulatory and homeostatic functions [3,4].

Cytokine TGF-β1 is a multifaceted protein [5,6,7,8] with a high impact on cellular responses to various brain injuries [9,10,11]. The bioavailability of TGF-βs is regulated by its release from cells, and also by extracellular mechanisms. TGF-β secreted from cells is tightly bound in a latent complex consisting of its dimeric pro-peptide (latency-associated peptide (LAP)) and a latent TGF-β-binding protein (LTBP). The complex of TGF-β, LAP, and LTBP is called the large latent complex (LLC). The TGF-β LLC is critical in modulating the action of the cytokine, and the controlled release of TGF-β from the ECM by activation is central to understanding TGF-β signaling. TGF-β1 exerts both neuroprotective and harmful effects, demonstrating that not a single particular action can be assigned to this cytokine, and its signaling role depends on multiple factors [10].

The neurological manifestations of acute HE include astrocytic impairment believed to dominate as a pathophysiological hallmark of the disease, and accompanied by impaired neurotransmission [2,12,13] leading to cognitive dysfunctions in chronic stages [14,15]. Disturbed neurotransmission involves altered astrocyte–neuron interactions that cause uneven distribution of ions and neurotransmitters, reflected in the imbalance between excitatory and inhibitory neurotransmission [1,2,16,17]. Recently, alterations in structural integrity and function of synapses were highlighted as elements contributing to neurotransmission disturbances [18]. Concomitantly, it was shown that exposure of cultured neurons to conditioned media from ammonia-treated cultured astrocytes resulted in decreased levels of synaptophysin, synaptotagmin, postsynaptic density protein 95 (PSD-95), as well as γ-Aminobutyric acid type A (GABA_A_) receptor and NR1 subunit of the N-methyl-D-aspartate (NMDA) receptor [19]. The correlation between increased thrombospondin 1 (TSP-1) level, TGF-βs cytokines activator [20,21], and normalization of ammonia-induced changes in the level of synaptic proteins was strongly suggested [19,22]. However, contrasting evidence delivered studies reporting that TSP-1 via TGF-β1 activation exacerbates the neuropathological status of ALF mice [23].

Subtle-to-severe leakage of the blood–brain barrier (BBB) in ALF animals, which has been frequently documented elsewhere, was postulated as the main route of liver-derived TGF-β1 increase in the brain leading to the activation of the neuronal receptor TGFβR2 signaling pathway [24,25], driving neuroinflammation and contributing to neurological decline [26].

Interestingly, TGF-β1 overexpression in neuronal culture and concomitant intracerebroventricular infusion of both TGF-β1 and anti-TGF-β1 in mice induce changes in the level of synaptophysin [27,28,29], some key subunits of α-amino-3-hydroxy-5-methyl-4-isoxazoleproprionic acid and NMDA receptors, and affect glutamatergic functions [30].

Thus, we hypothesized that changes in the TGF-β1 level in the course of acute HE may likewise modulate the expression of synaptic proteins and affect neurotransmission. In this study, we address the question of whether a selectively reduced circulating active form of TGF-β1 in mice injected with anti-TGF-β1 affects the expression and distribution of pre- and post-synaptic proteins and electrophysiological parameters, thereby contributing to glutamatergic neurotransmission impairment observed in the ALF mice.

To recreate the effect of reduced TGF-β1 in the ALF pathology, we administered the TGF-β1 neutralizing antibody intraperitoneally to control mice. We used a well-characterized azoxymethane (AOM) mice model of acute HE [18,31,32] and analyzed the effects induced by TGF-β1 reduced signaling in both models at the level of expression and distribution between the cytosolic and membrane fraction of pre- and post-synaptic proteins. Basic physiological parameters (field potential amplitude and ratio, long-term potentiation (LTP)) were recorded in mice after anti-TGF-β1 administration.

Since both up- and down-regulation of TGF-β1 signaling differently modulates tightness of BBB [33] were repeatedly but differently addressed in a plethora of contexts, we also aimed to clarify the impact of a decrease in TGF-β1 on BBB intactness and functionality. Thus, we analyzed the effects of anti-TGF-β1 in the presence of ammonia, a key factor in HE pathology, on the permeability of brain endothelial cells (rat brain endothelial cell line—RBE-4) and protein expression of critical tight junction proteins in the AOM mice brain homogenates.

## 2. Results

### 2.1. Blood Morphology and Biochemical Characteristics of the AOM Model

**Figure 1:**
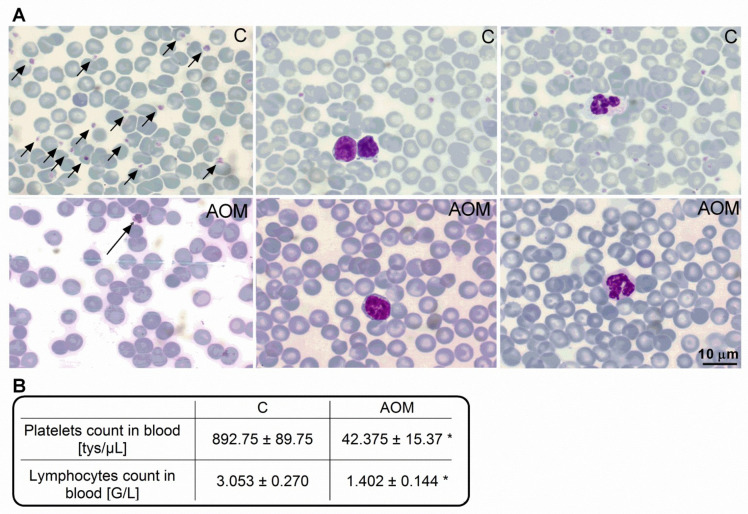
Analysis of peripheral blood parameters. (**A**) Representative images of blood morphotic elements in control (C; **upper**) and azoxymethane (AOM) (**bottom**) mice (from left: thrombocytes indicated with arrows; lymphocytes; neutrophils). (**B**) Platelet and lymphocyte count in the blood of control and AOM mice. Results are mean ± SEM. *n* = 4; * *p* < 0.01 vs. control, *t*-test.

Cytological analysis of blood smears showed unchanged morphology of lymphocytes and thrombocytes (Figure 1A). The quantitative analysis revealed a reduction by ~55% and ~95% in the total number of lymphocytes and platelets, respectively, in the AOM mice compared to controls (Figure 1B). Furthermore, neutrophils predominated in the manual leukogram. Results indicate the occurrence of lymphocytopenia and thrombocytopenia in the AOM mice.

### 2.2. Endothelial Cells Monolayer Permeability Measurement and the Expression of Blood–Brain Barrier Proteins in the Frontal Cortex Homogenates from AOM and Anti-TGF-β1 Mice

**Figure 2:**
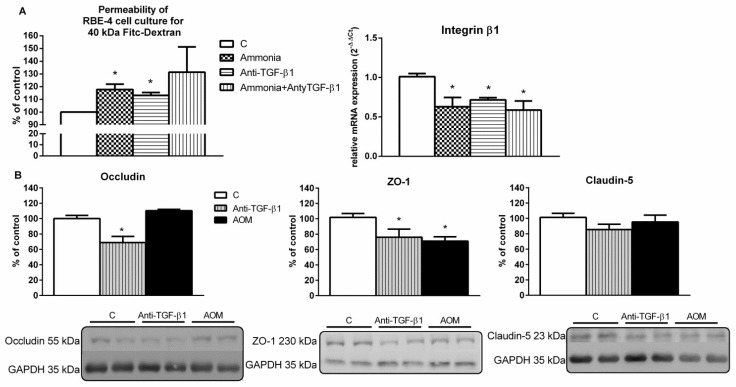
The effect of transforming growth factor 1 (TGF-β1) neutralizing antibody on the endothelium. (**A**) Endothelial cells monolayer (in vitro model of blood-brain barrier) permeability for 40 kDa fluorescein-5-isothiocyanate (FITC)-dextran (**left**) and integrin β1 mRNA expression level (**right**) after exposure for 24 h to TGF-β1 neutralizing antibody (Anti-TGF-β1; 2.5 µg/mL) and/or ammonium chloride (ammonia; 5 mM); *n* = 5, * *p* < 0.05 vs. control, one-way ANOVA, Dunnett’s post hoc test. (**B**) Protein levels of occludin, ZO-1, and claudin-5 in the frontal cortex homogenates from anti-TGF-β1 and AOM mice with representative immunoblots. Results are the mean ± SEM. *n* = 4; * *p* < 0.05 vs. control, one-way ANOVA, Dunnett’s post hoc test.

Endothelial cells exposed to ammonia and/or TGF-β1 neutralizing antibody for 24 h resulted in increased permeability of RBE-4 monolayer by ~17% and ~13%, respectively. The expression of integrin β1mRNA for all treatments was decreased by ~37%, ~29%, and ~42%, respectively (Figure 2A).

The protein level of occludin and tight junction protein-1 (ZO-1) in the brain cortex homogenates of anti-TGF-β1 mice was decreased by ~30% and ~25%, respectively, while the level of claudin 5 presented a downward trend. In the AOM mice brain cortex, a ~30% decrease in ZO-1 protein level was observed (Figure 2B). Results suggest that a deficit of TGF-β1 may affect BBB tightness, as documented in experiments in vitro, and contribute to BBB integrity alteration as observed in the frontal cortex homogenates from anti-TGF-β1 and AOM mice.

### 2.3. Parameters of TGF-β1 Signaling in Serum and Frontal Cortex Homogenates from AOM and Anti-TGF-β1 Mice

**Figure 3:**
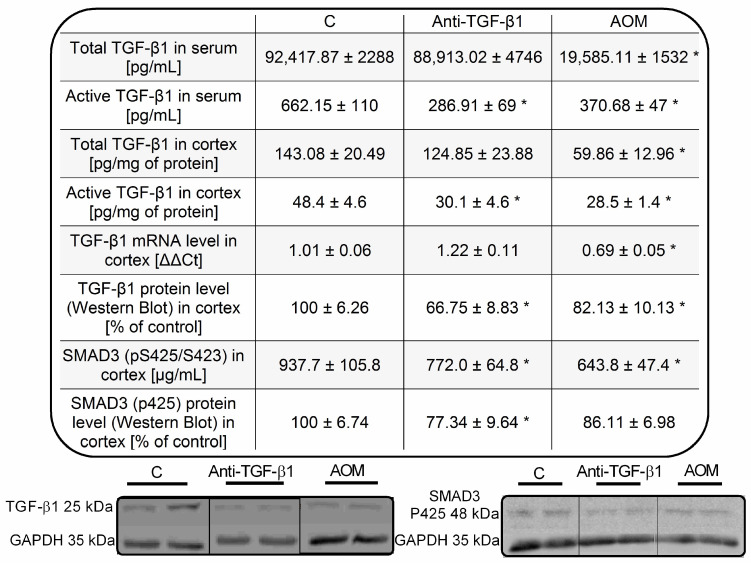
The TGF-β1 signaling parameters in serum and frontal cortex homogenates from anti-TGF-β1 and AOM mice. Results are the mean ± SEM. *n* = 5–6, *n* = 4 for SMAD3 P425 Western blot experiment; * *p* < 0.05 vs. control, one-way ANOVA, Dunnett’s post hoc test.

The level of total TGF-β1 was unaltered while TGF-β1 active form was reduced by ~57% in the serum of mice after TGF-β1 neutralization (Figure 3). The above parameters were reduced in the AOM mice by ~79% and ~44%, respectively (Figure 3). The level of total TGF-β1 was unaltered in the brain cortex homogenate of anti-TGF-β1 mice, while TGF-β1 cytokine active form was reduced by ~38%. In the brain cortex of AOM mice, the level of total and active TGF-β1 was reduced by ~58% and ~41%, respectively. The results corroborate with Western blot analysis, in which content of TGF-β1 active form in homogenates from anti-TGF-β1 and AOM mice cortex was reduced by ~33% and ~18%, respectively. Gene expression of TGF-β1mRNA was reduced by ~32% in the AOM model. A tendency to increase TGF-β1mRNA in the anti-TGF-β1 mice cortex was observed. The level of phosphorylated mothers against decapentaplegic homolog 3 (SMAD3) protein in brain cortex homogenates of anti-TGF-β1 and AOM mice was reduced by ~18% and ~31%, respectively (Figure 3). Results documented a decrease of TGF-β1 active form in both serum and brain cortex homogenates reflected in the reduced signaling in anti-TGF-β1 and AOM models.

### 2.4. Expression and Distribution of Synaptic Proteins in Frontal Cortex Homogenates from AOM, and Anti- TGF-β1 Mice

**Figure 4:**
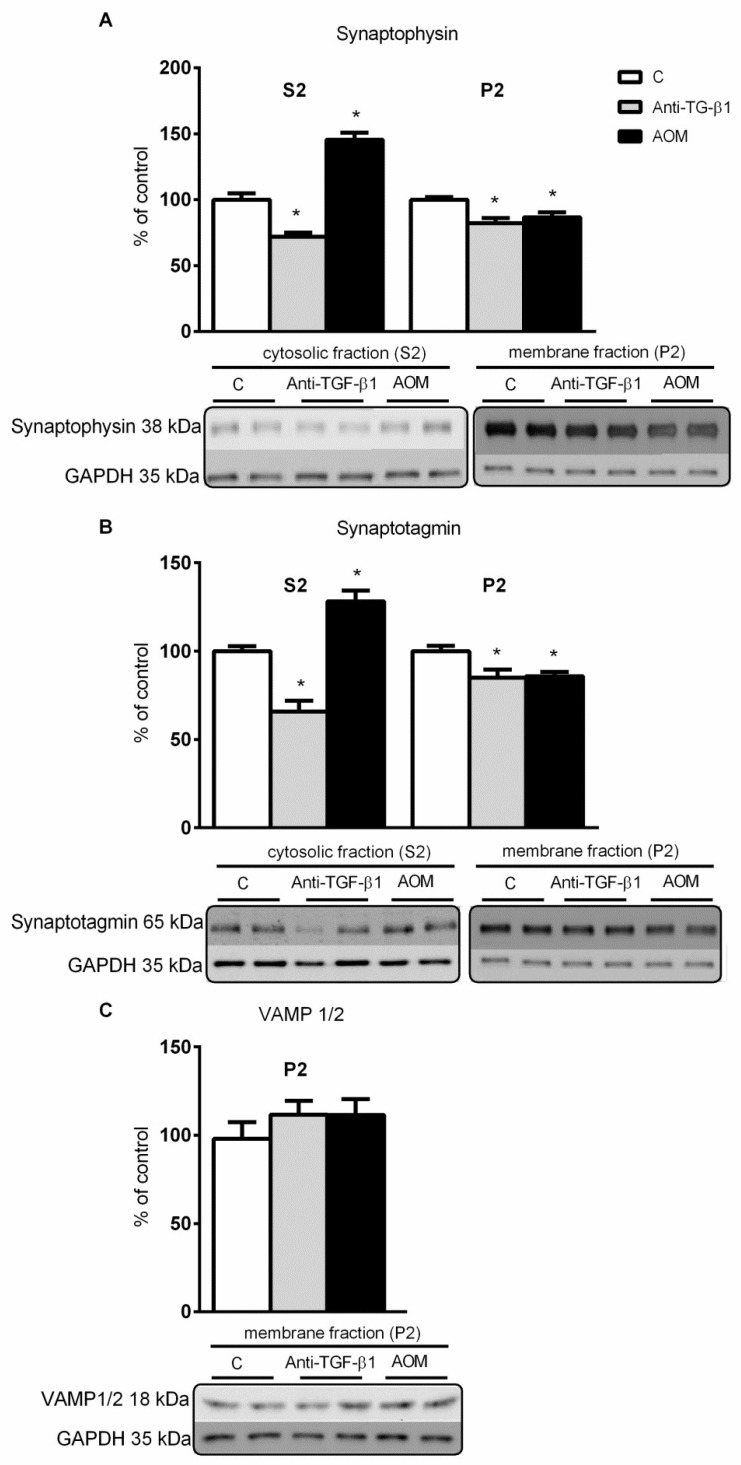
Expression and distribution of presynaptic proteins in frontal cortex from AOM, and anti-TGF-β1 mice. (**A**–**C**) Level of synaptophysin, synaptotagmin (in cytosolic and membrane fraction), and VAMP 1/2 (in membrane fraction) in frontal cortex from mice after TGF-β1 neutralization and AOM injection with representative immunoblots. Results are the mean ± SEM. n = 6 for synaptophysin and synaptotagmin, *n* = 4 for VAMP 1/2, * *p* < 0.05 vs. control, one-way ANOVA, Dunnett’s post hoc test.

An increase in both synaptophysin and synaptotagmin in cytosolic (by ~45% and ~37%, respectively), and a decrease in a membrane fraction (by ~14% and ~15%, respectively) were confirmed in the AOM model (Figure 4A,B). A decrease in analyzed proteins in both fractions (for cytosolic by ~28% and ~34%, and membrane by ~18% and ~15%, respectively) occurred in anti-TGF-β1 mice (Figure 4A,B). Docking-related vesicle-associated membrane proteins (VAMP 1/2) level was unaltered in both models (Figure 4C).

**Figure 5:**
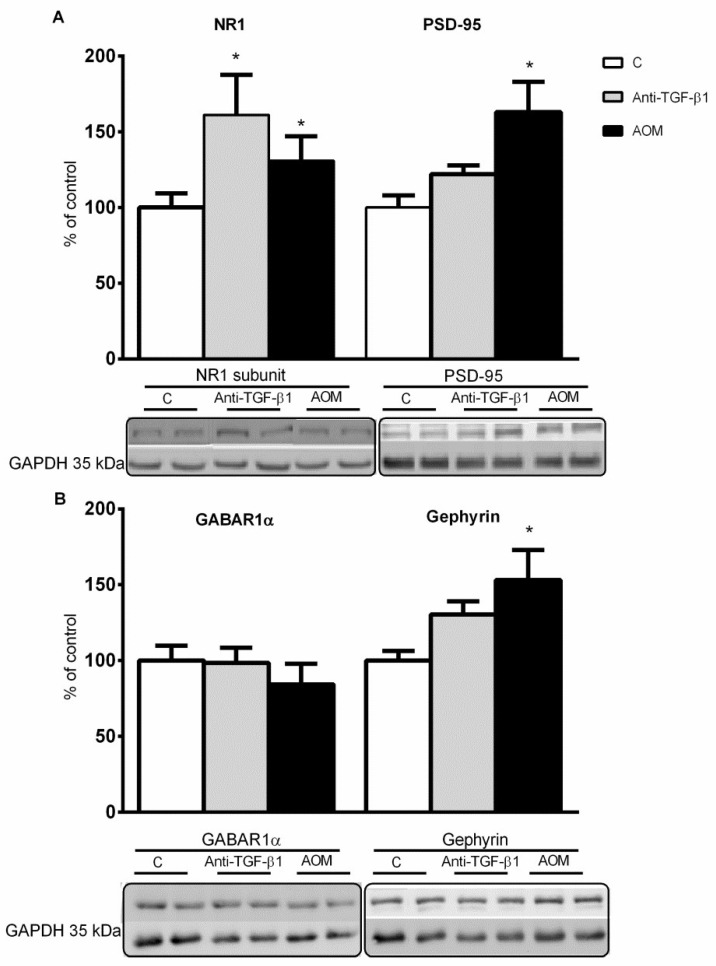
Expression of post-synaptic proteins in frontal cortex from AOM, and anti-TGF-β1 mice. (**A**,**B**) Level of NR1 subunit, PSD-95, GABAR1α, and gephyrin in membrane fraction in frontal cortex from mice after TGF-β1 neutralization and AOM injection with representative immunoblots. Results are the mean ± SEM. *n* = 6 for NR1 and PSD-95, *n* = 4 for GABAR1α and gephyrin, * *p* < 0.05 vs. control, one-way ANOVA, Dunnett’s post hoc test.

In turn, an increased level of postsynaptic proteins involved in glutamatergic signalization—NR1 subunit of NMDA receptor and PSD-95—in the brain cortex membrane fraction by ~29% and ~63% for the AOM model and by ~61% and a tendency toward the increase in mice after TGF-β1 neutralization, respectively, was observed (Figure 5A). Moreover, post-synaptic proteins involved in GABAergic transmission—subunit of GABA_A_ receptor 1α (GABAR1α)—was unchanged, but the gephyrin level was elevated by ~53% in AOM model membrane fractions and shows a strong tendency to increase in anti-TGF-β1 mice (Figure 5B).

### 2.5. Electrophysiological Responses of Frontal Cortical Slices from Anti-TGF-β1 Mice

**Figure 6:**
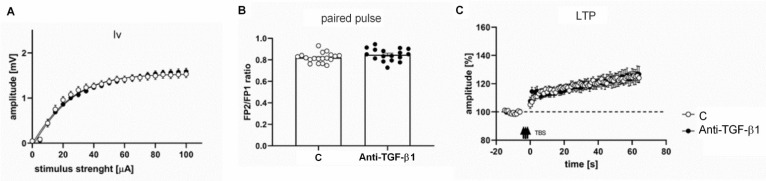
Electrophysiology of cerebrocortical slices from anti-TGF-β1 mice. (**A**) The amplitudes of field potentials of control and anti-TGF-β1 mice. (**B**) Summary quantification of the average paired pulse ratio. (**C**) Long-term potentiation (LTP) analysis, arrows denote the theta burst insets, 18 measurements for control and 15 for anti-TGFβ1. Results are the mean ± SEM. *n* = 4.

Analyses of FPs recorded in brain slices obtained from anti-TGF-β1 mice showed no changes in the relationship between stimulus intensity and FP amplitude (input–output curve) compared with the slices obtained from control animals (Figure 6A,B). LTP recorded in layer II/III was unaltered as well (Figure 6C).

## 3. Discussion

We analyzed the effect of TGF-β1 reduced signaling on the expression of synaptic transmission proteins and electrophysiology parameters in the frontal cortex of mice, confronted with data obtained from ALF mice, which presented a thrombocytopenia-linked reduction in circulating cytokine TGF-β1 concentration. In our study, AOM mice, apart from reproducing standard behavioral and biochemical blood parameter alterations of acute HE (data not shown), presented lymphopenia and thrombocytopenia (Figure 1B), frequently reported in patients with ALF [34] and cirrhosis [35]. From a clinical standpoint, thrombocytopenia in liver diseases [36] is often associated with splenomegaly [37,38] but may also include reduced thrombopoietin released from the impaired liver, and in this way affects platelet production [39,40]. Lymphopenia is recognized at the early stages of cirrhosis and can be associated with abnormal production of new T lymphocytes and enhanced apoptosis [41,42]. Additionally, blood analysis of AOM mice with the predominant number of neutrophils in the manual leukogram indicates the increase of neutrophil-to-lymphocyte ratio (NLR), a clinically relevant index associated with severity and mortality of patients with liver diseases [43,44].

The visible changes in hepatic histopathology (Supplementary 1A) and a five-fold increase of active TGF-β1 cytokine in the AOM liver tissue homogenate (Supplementary 1B) were consistent with observations from studies conducted by the group of Prof. DeMorrow [24]. However, from our perspective, the significant increase of TGF-β1 in the liver does not reflect elevated plasma levels of this cytokine, as indicated in the above-mentioned study. Inversely, serum TGF-β1 of both total and active form was significantly decreased, in line with a reduced number of platelets in AOM mice. Thus, we assumed that a reduced count of platelets, considered as a reservoir of secreted plasma TGF-β1, determines the level of inactive form of TGF-β1 measured in our model.

Importantly, platelets contain approximately 40–100× more TGF-β1 than other cells [3] and release the latent form of TGF-β1 in response to activation [3,45,46]. The activation of latent TGF-β1 is independent of cytokine release and may process in diverse modes that suggest cell type-selective or tissue-selective mechanisms depending on the signaling context [47]. Combined interaction with integrins (αvβ1, αvβ6, and αvβ8) and/or various proteases confer physiological activation of latent TGF-β1. The extracellular matrix (ECM) proteins thrombospondin and fibronectin, as well as fibrillin-1, also direct TGF-βs activation, but their roles are less clear [48,49,50]. In turn, in response to tissue injury, bacterial infection, or alteration in blood flow, platelets become activated, interact with the immune cells, and contribute to cellular responses to maintain vascular homeostasis [51,52].

The TGF-β1 level changes in the blood are likely to affect this cytokine concentration in the brain [24,25,26]. At present, however, it is unknown whether there is any flux of TGF-β1 across the vascular endothelium (in either direction) which could lead to equilibration between tissue and blood level of TGF-β1. It is worth noting, in this context, that TGF-β, secreted in a latent form, binds to αv integrins, resulting in TGF-β activation through release from the latent binding protein [53,54]. However, it is not entirely clear whether this is valid for αvβ1 integrin analyzed in this study [55].

In our experiments, treatment of brain endothelial cells, an in vitro model of BBB, with anti-TGF-β1 acting alone or in the presence of ammonia increased permeability of the monolayer, an effect accompanied by the reduced αvβ1 integrin mRNA level (Figure 2A). The observation is in line with the observed interference with β1-integrin-collagen IV adhesion in confluent endothelial monolayer cells that facilitate the reorganization of the inter-endothelial tight junction complex components (e.g., claudin-5, occludin, and ZO-1) and increased monolayer permeability [56]. Importantly, the loss of integrin β1 expression in the cerebral microvessels after mild cerebral artery occlusion coincides with increased BBB permeability and edema development [56]. In line with the above, other studies have shown that anti-TGF-β1 antibody or a TGF-β type I receptor antagonist (SB431542) treatment of pericytes and mouse brain capillary endothelial cells co-culture increases the permeability of sodium fluorescein and the accumulation of rhodamine in endothelial cells [57]. Furthermore, it was shown that conditioned media from oligodendrocyte precursor cells that secrete TGF-β1, added to RBE4 cells, increase cell monolayer tightness through elevating the expression of tight junction proteins (ZO-1, occludin, claudin-5), and the effect was blocked after TGF-β receptor signaling inhibition [58].

Results from our laboratory and others repeatedly documented increased permeability of the BBB and brain edema in the AOM mice [25,59,60,61]. Here, documented BBB impairment revealed by the decrease of tight junction proteins (Figure 2B), accompanied by a significant decrease of active form TGF-β1, as it was measured in brain tissue of AOM mice, reflected further in TGF-β1 decreased signaling, revealed by the reduced protein level of TGF-β1 and phosphorylated intracellular effector protein SMAD3, after its recruitment to the TGF-βRII receptor.

Intraperitoneal administration of TGF-β1-neutralizing antibody resulted in reduced TGF-β1 active form and diminished signaling in the frontal cortex of mice (Figure 3). Since TGF-β1 neutralizing antibody interacts solely with an active form of TGF-β1 and cannot affect transcription of TGF-β1, observed changes in serum TGF-β1 in the anti-TGF-β1 mice strictly reflect cytokine level measured in the brain tissue. It should be highlighted that both mice models used in this study present comparable concentrations of an active form TGF-β1 that is important regarding analysis related to the neurotransmission.

Recent findings indicated the involvement of TGF-βs signaling pathways in the modulation of both excitatory and inhibitory synaptic transmission in the adult mammalian brain [62]. Deficits in TGF-β signaling lead to an increase of GABA neurotransmission in the midbrain and the hippocampus [63,64] by affecting the expression of the GABA_A_ receptor α6 subunit [65]. Importantly, changes in GABAergic neurotransmission are attributed to a decrease in canonical activity of the TGF-β-SMAD3 pathway, as was demonstrated, for example, in the SMAD3 deficient mice model [66]. Moreover, the scaffold protein gephyrin anchors GABA_A_ receptors to the cytoskeleton [67]. This protein interacts with different molecules to modulate synapse formation and plasticity likely to be activated by the Erk1/2, GSK3β, and CDKs signaling pathways, which are similar to the activation of the TGF-β-dependent SMAD3 pathway [64,68,69]. In this study, we showed an unaltered level of GABA receptor 1α subunit and an elevated amount of gephyrin in membrane fractions from the brain cortex of AOM mice and a strong tendency toward an increase in anti-TGF-β1 tissue (Figure 5B). Several reports indicated increased GABAergic tone in acute liver failure models [70,71,72,73] or in vivo studies in which high ammonia concentrations were used [74]. Nevertheless, the GABA_A_ receptor proteins are unchanged in either HE patients or experimental HE [75]. The effect might be explained by the brain accumulation of benzodiazepines and neurosteroids, the increased cerebral cortical release of GABA, or the action of high concentrations of ammonia by enhancing the ability of GABA to depress neuronal activity and its synergistic interaction with benzodiazepine receptor ligands [2,76]. It is assumed that increased GABAergic tone contributes to more pronounced imbalance resulting from altered glutamatergic neurotransmission.

In this context, it was previously reported that decreased levels of TGF-β1 reduce the level of glutamate transporters GLT-1 (EAAT2) and GLAST (EAAT1), and glutamate uptake in the mouse hippocampus [77]. So far, the studies demonstrating directly the effect of TGF-β1 deficiency on the level of the postsynaptic protein components were inconclusive and affected by the experimental conditions [30,65,66,77]. TGF-β1-deficient mice presented elevated NMDA receptor subunit N2B-mediated calcium signal in response to extrasynaptic glutamate receptor stimulation, but unaltered levels of post-synaptic proteins were observed [77]. It is worth adding that a significant increase of NR1 and NR2 subunit expression in the hippocampus of mice with TGF-β1 overexpression was concomitantly demonstrated [30].

The anticipated contribution of TGF-β1 in previously reported dysregulated distribution and content of critical synaptic proteins and altered electrophysiological parameters in AOM mice [18] only partially reflects changes observed in the present study in the anti-TGF-β1 mice. Reduced levels of synaptophysin and synaptotagmin in the cytosolic fraction were only observed in anti-TGF-β1 and not in AOM mice. In turn, decreased levels of proteins in the membrane fraction coincided with AOM-induced changes (Figure 4). The results are in line with the results presented by other groups showing the TGF-β1 decrease-dependent reduction in synaptic proteins: drebrin, synaptophysin, and PSD-95 after intracerebroventricular infusion of amyloid-β oligomers in mice, and after treatment of cultured astrocytes with amyloid-β oligomers [28]. It should be noted that the administration of TGF-β1 together with amyloid-β oligomers normalized the expression of synaptic proteins. Accordingly, a study on neural cultures treated with TGF-β1 showed that it results in increased synaptophysin [27,29].

Our results suggest that decreased TGF-β1 signaling observed in the acute HE may aggravate the deficiency of synaptophysin and synaptotagmin in the membrane fraction, but does not explain the mechanism of impaired protein distribution. The changes are likely to reflect a decreased response to presynaptic activity. In the AOM model, the compensatory mechanism seems insufficient; thus, a reduction in LTP and field potential amplitudes was recorded [18], which can result from other mechanisms affecting glutamatergic transmission. However, the identification of other factors influencing LTP [78,79] is beyond the scope of this study. In the neutralization model, changes in synaptic protein expression level are not translated into changes in electrophysiological parameters. It was suggested that LTP was inhibited in SMAD3-deficient mice resulting from the activation of GABA receptors [66]. The other explanation may be linked with incomplete inactivation of SMAD3. However, this possibility cannot reasonably explain effects induced by ~30% reduction of TGF-β1, the main activator of SMAD3 receptor in both models used in the present study. In the context of ALF, it was demonstrated that intraperitoneal administration of an antibody neutralizing the TGF-β1 improves the neurological status and increased the expression of Gli1, a protein showing neuroprotection in the HE [24]; however, the exact mechanism was not proposed.

It is worth noting that the involvement of TGF-βs was implicated in the pathology of neurodegenerative diseases [80]. It was proposed that impaired TGF-β signaling in neurons contributes to β amyloid accumulation, microglial activation [81], and neurodegeneration [82]. The diminished function of the TGF-β pathway reduced neurofibrillary tangle formation [83] and relieved tau pathology, indicating neuroprotective properties of TGF-β1. Importantly in this context, conflicting results have also been reported. Thus, the exact contribution of the TGF-β1 signaling in neurodegenerative disorders is still unclear.

In conclusion, reduced concentration of active TGF-β1 resulting in TGF-β1 decreased signaling in the frontal cortex of mouse brain may aggravate the deficiency of synaptophysin and synaptotagmin content in the membrane fraction, determining the efficiency of vesicle trafficking to the membrane, observed in the acute model of HE, but does not explain the mechanism of impaired protein distribution. Moreover, due to different electrophysiology responses recorded in both models, it might be concluded that decreased TGF-β1 signaling, evoked by thrombocytopenia during ALF, is not critical in the neurotransmission disturbances observed in acute HE.

## 4. Materials and Methods

### 4.1. Animal Models

All experiments were performed with the agreement of the local animal ethical committee in Warsaw (498/2017) following EC Directive 86/609/EEC. Male C57Bl6 mice (an animal colony of the Mossakowski Medical Research Centre, Polish Academy of Sciences in Warsaw), bodyweight 25.0 ± 5.1 g, were subjected to a hepatotoxic insult by single i.p. injection of AOM (100 mg/kg b.w. A5486, Sigma Aldrich, Poznań, Poland) or to neutralize cytokine—anti-TGF-β1 neutralizing antibody (1 mg/kg b.w. AB-101-NA, R&D Systems, Bio-Techne, Minneapolis, MN, USA). All requirements for appropriate treatment of animals have been met (free access to water and chow, housed in constant temperature, humidity, and 12 h light–dark cycling). Unless otherwise stated, experiments were performed after the first symptoms of coma in mice in the AOM model—selected according to neurological tests [18]—and at the same time after neutralization.

### 4.2. Study Design and Animal Groups

**Figure 7:**
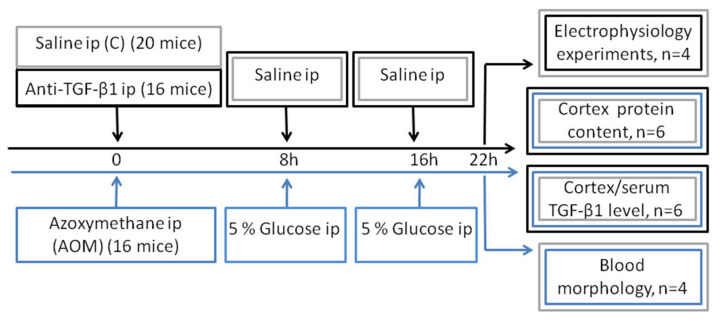
Graphical representation of experimental protocol.

Decent planning of the experiments required the creation of three independent groups of animals: control (C, 20 mice), acute liver failure (AOM, 16 mice), and mice with TGF-β1 neutralization (anti-TGF-β1, 16 mice). All further experiments were performed approximately 22 h after administration of AOM/anti-TGF-β1 antibody/first saline dose for control. Electrophysiological experiments were carried out on four control and four anti-TGF-β1 mice. The measurement of TGF-β1 signaling parameters in serum and frontal cortex homogenates was performed on five control, six AOM, and six anti-TGF-β1 mice. Six mice from each group were used in the expression and distribution of proteins in the frontal cortex study (detailed group sizes for the measurement of each protein are provided in the description of the figures). Analysis of peripheral blood parameters was conducted on four control and four AOM mice (Figure 7). Except electrophysiological analysis, the samples were entirely blinded for investigators.

### 4.3. Analysis of Biochemical Parameters

Quantitative and qualitative microscopic evaluation of peripheral blood was performed by AlabVet (ALAB plus Sp. z o.o., Warsaw, Poland). Briefly, a blood smear was taken immediately after decapitation of the animals, transferring a blood drop directly to a microscope slide, and a smear was made so that the dense body blended smoothly into the monolayer area. Then, the slides were air-dried (24 h). After staining (May–Grünwald–Giemsa and the rapid staining kit for blood and bone marrow smears—RapiHem), the slides were analyzed for the thrombocyte and lymphocyte counts and morphological assessment of cells on an Axiolab 5 microscope (Zeiss, Jena, Germany).

### 4.4. TGF-β1 Level Determination

To measure total and active TGF-β1 content in the blood, the serum was isolated as described in kit guidelines (Quantikine ELISA Mouse TGF-b1 MB100B R&D Systems, Bio-Techne, Minneapolis, MN, USA). Briefly, blood was collected and allowed to clot at room temperature for 40 min and centrifuged for 20 min at 2500× *g*. To activate latent TGF-β1 to the immunoreactive form, 1 N HCl was added to samples for 10 min, then neutralized by 1.2 N NaOH/0.5 M HEPES. The samples after activation were diluted 60-fold, while the non-activated samples were diluted 5-fold. Optical density was measured spectroscopically at a wavelength of 450 nm with wavelength correction at 570 nm, and the concentration was calculated from the standard curve.

To measure total and active TGF-β1 content in the brain cortex, tissue was immediately isolated on ice, homogenized in buffer (20 mM Tris-HCl, pH 6.8; 137 mM NaCl; 2 mM EDTA; 1% Triton X-100; 0.5 mM DTT; 0.5 mM PMSF; Phosphatase Inhibitor cocktail 1:100; Protease Inhibitor Cocktail 1:200), and centrifuged at 12,000× *g* for 10 min. Protein concentration was carried out by the BCA Protein Assay method from Thermo Scientific (Pierce, Rockford, IL, USA). Total TGF-β1 concentration was measured after acid activation as described for serum determination in the previous paragraph (no dilution) using R&D Systems Kit. Legend Max Free Active TGF-β1 ELISA Kit (437707; BioLegend Inc., San Diego, CA, USA) was used to determine the level of the active form. Absorbance was measured at a wavelength of 450 nm with wavelength correction at 570 nm, and the concentration was calculated from the standard curve and converted into milligrams of protein.

Colorimetric SMAD3 (pS423/S425) ELISA Kit (ab 186038, Abcam, Cambridge, UK) was used to determine the endogenous level of SMAD3 phosphorylated at Ser423/425 in brain cortex homogenates. SMAD3 is a transcriptional modulator activated by TGF-β1 and activin type 1 receptor kinase. The same cortex homogenates as in the TGF-β1 content experiment were used in 4-fold dilution. Signal intensity was measured at 450 nm and the concentration was calculated from the standard curve and converted into milligrams of protein.

### 4.5. Endothelial Cell Line Culture

Immortalized RBE-4 cells were cultured on the Minimum Essential Media (MEM)/Gibco Ham’s F-10 Nutrient Mixture (Thermo Fisher Scientific, Waltham, MA, USA) with the addition of 10% Fetal Bovine Serum (FBS), Basic Fibroblast Growth Factor (bFGF) and Gentamicin (all from Thermo Fisher Scientific, Waltham, MA, USA). The cells prepared at a density of 25,000/cm^2^ were cultured at 37 °C in an atmosphere of 95% O_2_ and 5% CO_2_ and after 4 days were used for further studies.

### 4.6. Permeability Assay

RBE-4 cells were seeded at 100,000 cells/insert density onto Transwell (Corning, New York, NY, USA) inserts 0.4 µm in diameter in a 24-well plate. The experiments were carried out after 3 days of cultivation when the cells covered the inserts with a tight monolayer. FBS content was gradually reduced to 1% 24 h before the experiment. Then, cells in test groups were exposed to 5 mM ammonium chloride (Sigma Aldrich, St. Louis, MO, USA) or anti-TGF-β1 neutralizing antibody (2.5 µg/mL, AB-101-NA, R&D Systems, Bio-Techne, Minneapolis, MN, USA) or both for 24 h. Monolayer permeability was measured using 40 kDa fluorescein-5-isothiocyanate (FITC)-labeled Dextran (Sigma Aldrich, St. Louis, MO, USA) at a concentration of 1 mg/mL. After 40 min incubation, 100 µL of samples were taken from the lower compartment, and fluorescence was measured (in duplicate) in a FLUOstar Omega (BMG LABTECH, Offenburg, Germany) at excitation/emission wavelength of 485 nm/520 nm. The amount of Dextran was expressed as % of the values obtained with control cells (untreated).

### 4.7. RNA Isolation and Real-Time PCR Analysis

Total RNA from the frontal cortex was isolated using TRI reagent (Sigma-Aldrich, Poznań, Poland), and reverse-transcribed using HighCapacity cDNA Reverse Transcription Kit (Applied Biosystems, Waltham, MA, USA).

Real-time PCR was performed with the ABI 7500 equipment (Applied Biosystems, Waltham, MA, USA). TaqMan Gene Expression Assay and primers for TGF-β1 and β-actin, as a control (Mm01178820_m1 and Mm00607939_s1, respectively), were purchased from Applied Biosystems. Expression was performed using 1 µL cDNA in the reaction of 10 µL. The real-time PCR reactions were performed at 95 °C for 10 min, followed by 40 cycles of 30 s at 95 °C and 1 min at 60 °C. The results of the analysis were calculated for the β-actin product, and results were calculated according to and expressed by, an equation (2^−ΔΔCt^) that gives the amount of target, normalized to an endogenous reference and relative to a calibrator. Ct is the threshold cycle for target amplification [84].

### 4.8. Immunoblotting Analyses

After decapitation, the brain was immediately removed and the cortex was isolated on ice. Tissue samples were homogenized in buffer (15 mM Tris-HCl, pH 7.6; 0.25 M sucrose, 1 mM DTT; 0.5 mM PMSF, Phosphatase Inhibitor Cocktail (1:100, P5726, Sigma Aldrich, Poznań, Poland); Protease Inhibitor Cocktail (1:200, P8340, Sigma Aldrich, Poznań, Poland)) and centrifuged at 1000× *g* for 10 min (4 °C) to isolate P1 fraction. The separated supernatant was centrifuged at 14,000× *g* for 20 min (4 °C) and cytosolic (S2) fraction was collected. The pellet, after buffer addition, was frozen as a membrane (P2) fraction. Protein concentrations were determined using a BCA Protein Assay (Thermo Scientific, Warsaw, Poland).

The content of proteins was assessed by immunoblotting as previously described [18,85]. First, 30 μg of protein from cytosolic or membrane fractions were diluted in Laemmli buffer (S3401 Sigma Aldrich, Poznań, Poland) and loaded in 12% SDS-PAGE gels, then transferred on to nitrocellulose membrane (1620112, Bio-Rad Laboratories GmbH, Munich, Germany). Membranes were blocked in 5% milk and incubated overnight at 4 °C with antibodies against synaptophysin (1:20,000, ab32127), ZO-1 (1:2000, 21773-1-AP), occludin (1:1500, 27260-1-AP), gephyrin (1:700, 12681-1-AP), GABARA1 (1:1500, 12410-1-AP, Proteintech Europe, Manchester, UK), claudin-5 (1:500, bs-1553R, Bioss Antibodies Inc. Woburn, MA, USA), TGF-β1 (1:300, ab9758, Abcam, Cambridge, UK) VAMP-1/2 (1:500, sc-20039), PSD-95 (1:200, sc-2894), synaptotagmin 1 (1:400, sc-136480, Santa Cruz Biotechnologies, Dallas, TX, USA), NR1 (1:200, PA5-55093, Thermo Fisher Scientific, Waltham, MA, USA), phospho SMAD3 PSER425 (1:500, SAB4503781, Sigma Aldrich, Poznań, Poland) in 1% milk and then, for 1 h in 1% milk with HRP-conjugated anti-rabbit IgG (1: 6000, 269A-1) or anti-mouse IgG (1:5000, A9917, Sigma-Aldrich, Poznań, Poland). The protein bands were visualized using the G-Box system (SynGene, Bengaluru, India). Data were expressed as fold change in fluorescent band intensity in relation to GAPDH (1:7500, HRP-60004, Proteintech, Manchester, UK), which was used as a loading control. Band intensity was analyzed using GeneTools software (SynGene, Cambridge, UK).

### 4.9. Electrophysiological Analysis

#### 4.9.1. Treatment of Animals and Brain Slice Preparation

The effects of TGF-β1 neutralization were studied ex vivo in the mouse frontal cortex slices. 18 h after anti-TGF-β1 i.p. injection, mice were anesthetized with isoflurane (Aerrane, Baxter). Brains were removed and immersed in an ice-cold artificial cerebrospinal fluid (ACSF) of the following composition (in mM): NaCl (130), KCl (5), CaCl_2_ (2.5), MgSO_4_ (1.3), KH_2_PO_4_ (1.25), NaHCO_3_ (26), and D-glucose (10), bubbled with a mixture of 95% O_2_ and 5% CO_2_. Frontal cortical slices (400 μm thick) were cut in the coronal plane using a vibrating microtome (Leica, Wetzlar, Germany). Slices were stored at 32 ± 0.5 °C.

#### 4.9.2. Field Potential Recording and LTP Induction

The slices were placed in the recording chamber of an interface type and were super-fused at 2.5 mL/min with warm (32 ± 0.5 °C), modified ACSF (see above). A concentric bipolar stimulating electrode (FHC, Inc., Bowdoin, ME, USA) was placed in cortical layer V. Stimuli of 0.033 Hz frequency and duration of 0.2 ms were applied using a constant-current stimulus isolation unit (WPI). Glass micropipettes filled with ACSF (2–5 MΩ) were used to record field potentials. Recording microelectrodes were placed in cortical layer II/III. The responses were amplified (EXT 10-2F amplifier, NPI), filtered (1 Hz–1 kHz), A/D converted (10 kHz sampling rate), and stored on PC using the Micro1401 interface and Signal 2 software (Cambridge Electronic Design Ltd., Cambridge, UK).

A stimulus-response (input–output) curve was made for each slice. To obtain the curve, stimulation intensity was gradually increased stepwise (15 steps; 5–100 μA). One response was recorded at each stimulation intensity. The recording was performed in standard ACSF with stimulation intensity adjusted to evoke a response of 30% of the maximum amplitude. LTP was induced by theta-burst stimulation (TBS). TBS consisted of ten trains of stimuli at 5 Hz, repeated 5 times every 15 s. Each train was composed of five pulses at 100 Hz. During TBS pulse duration was increased to 0.3 ms.

### 4.10. Statistical Analyses

The number of groups was as shown in the description of the figures, in repetitions. A *t*-test for two groups analysis or an ANOVA followed by Dunnett’s post hoc tests were used. In all figures, the data represent the mean ± standard error of the mean (mean ± SEM); * *p* < 0.05. For statistical analyses, GraphPad Prism 7 (GraphPad Software Inc., San Diego, CA, USA) was used.

## Data Availability

Data available on request.

## Supplementary Materials

The following supporting information can be downloaded at: https://www.mdpi.com/article/10.3390/ijms23031081/s1.

## Abbreviations

ALF	Acute liver failure
AOM	Azoxymethane
BBB	Blood–brain barrier
CNS	Central nervous system
DMEM	Dulbecco’s Modified Eagle’s Medium
FBS	Fetal bovine serum
FITC	Fluorescein-5-isothiocyanate
FPs	Whole-cell field potentials
GABA	γ-aminobutyric acid
GABAA	γ-aminobutyric acid type A receptor
GABAR1α	subunit of GABA_A_ receptor 1α
GAPDH	Glyceraldehyde 3-phosphate dehydrogenase
HE	Hepatic encephalopathy
LAP	Latency-associated peptide
LLC	Large latent complex
LTBP	Latent TGF-β-binding protein
LTP	Long-term potentiation
MBEC4	Mouse brain capillary endothelial cell line
NMDA	N-methyl-D-aspartate
PBS	Phosphate-saline buffer
PSD-95	Postsynaptic density protein 95
RBE-4	Rat brain endothelial cell line
RT	Room temperature
SMAD3	Mothers against decapentaplegic homolog 3 protein
TBS	Theta-burst stimulation
TGF-β1	Transforming growth factor beta 1
TSP-1	Thrombospondin 1
VAMP 1/2	Vesicle-associated membrane proteins
WT	Wild-type
ZO-1	Tight junction protein 1 or Zonula occludens-1
