# Supplementary material for: The Effect of TGF-β1 Reduced Functionality on the Expression of Selected Synaptic Proteins and Electrophysiological Parameters: Implications of Changes Observed in Acute Hepatic Encephalopathy
Source: Int J Mol Sci. 2022 Jan 19;23(3):1081. doi: 10.3390/ijms23031081 (PMC8835518; doi:10.3390/ijms23031081)
Supplement: Supplementary file 1 [file ijms-23-01081-s001.zip › ijms-1523759-supplementary.pdf]

A

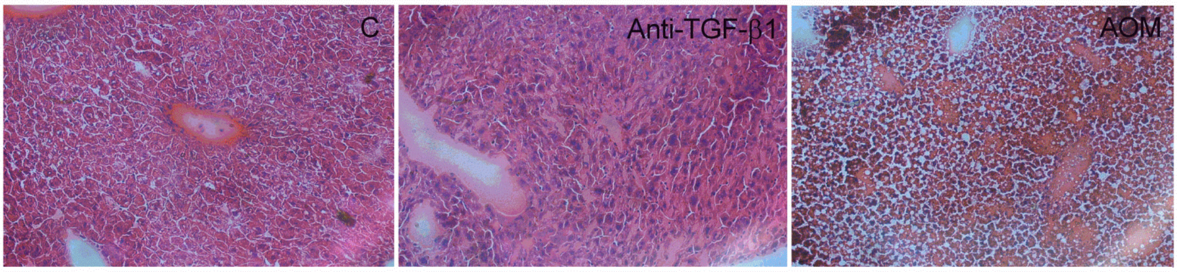

B

	C	AOM
Total TGF-β1 in liver [pg/mL]	85.79 ± 12.10	751.66 ± 204.09 *

Figure S1. (**A**) Liver sections of control, anti-TGF-β1, AOM mice. H&E staining, magnification: ×200. (**B**) The total concentration of TGF-β1 measured in liver homogenates from control and AOM mice. Results are the mean ± SEM. $n = 3$, * $p < 0.05$, t-test.

Materials and Methods

Histological Evaluation of Liver Sections

Liver sections were stained by the standard hematoxylin and eosin method for histological evaluation. Briefly, the liver tissues fixed in ice cold acetone were cut into 6 μm sections on a cryostat. The slides were then washed with a gradient of ethanol starting at 99% and ending with 70% and water. The sections were then stained with hematoxylin (Sigma Aldrich, St. Louis, MO, USA) solution and rinsed under running water for 10 min. After 4 min of incubation in eosin (Sigma Aldrich, St. Louis, MO, USA) solution, sections were washed with an inverted ethanol gradient starting at 70% and ending at 99%. The sections were sealed in Canadian balsam and analyzed using an Olympus IX71 Inverted Fluorescence Motorized Microscope (Olympus Corporation, Tokyo, Japan).

Measurement of Total TGF-β1 Concentration in Liver

To measure total TGF-β1 content in the liver, tissue was immediately isolated on ice, homogenized in buffer (PBS; Phosphatase Inhibitor cocktail 1:100; Protease Inhibitor Cocktail 1:200), and centrifuged at 12,000× *g* for 10 min. Protein concentration was carried out by the BCA Protein Assay method from Thermo Scientific (Pierce, Rockford, IL, USA). Total TGF-β1 concentration was measured using Quantikine ELISA Mouse TGF-β1 KIT (MB100B R&D Systems, Bio-Techne, Minneapolis, MN, USA) after acid activation. 1 N HCl was added to samples for 10 min, then neutralized by 1.2 N NaOH/0.5 M HEPES. The samples after activation were diluted 20-fold, optical density was measured spectroscopically at a wavelength of 450 nm with wavelength correction at 570 nm. Protein concentration was carried out by the BCA Protein Assay method from Thermo Scientific (Pierce, Rockford, IL, USA). TGF-β1 concentration was calculated from the standard curve and converted into milligrams of protein.
